# BrainCheck – a very brief tool to detect incipient cognitive decline: optimized case-finding combining patient- and informant-based data

**DOI:** 10.1186/s13195-014-0069-y

**Published:** 2014-11-24

**Authors:** Michael M Ehrensperger, Kirsten I Taylor, Manfred Berres, Nancy S Foldi, Myriam Dellenbach, Irene Bopp, Gabriel Gold, Armin von Gunten, Daniel Inglin, René Müri, Brigitte Rüegger, Reto W Kressig, Andreas U Monsch

**Affiliations:** Memory Clinic, University Center for Medicine of Aging Basel, Felix Platter-Spital, Schanzenstrasse 55, 4031 Basel, Switzerland; Centre for Speech, Language and the Brain, Department of Psychology, University of Cambridge, Downing Street, Cambridge, CB2 3EB UK; Department of Mathematics and Technology, University of Applied Sciences, Koblenz, Joseph-Rovan-Allee 2, 53424 Remagen, Germany; Department of Psychology, Queens College and The Graduate Center of The City University of New York, 65-30 Kissena Blvd., NSB-E318, Flushing, NY 11367 USA; Center for Gerontology, University of Zurich, Sumatrastrasse 30, 8006 Zurich, Switzerland; Currently: Center for Medical Education, Max-Daetwyler-Platz 2, 3014 Berne, Switzerland; Department of Geriatrics, Town Hospital Waid, Tièchestrasse 99, 8037 Zurich, Switzerland; Department of Internal Medicine, Rehabilitation and Geriatrics, University of Geneva, Ch. du Pont-Bochet 3, 1226 Thônex, Switzerland; Department of Old Age Psychiatry, University of Lausanne, Route du Mont, 1008 Prilly, Switzerland; Department of Geriatrics, General Hospital, Rorschacher Strasse 94, 9000 St. Gallen, Switzerland; Department of Neurology, University Hospital, Inselspital, 3010 Berne, Switzerland; University Center for Medicine of Aging Basel, Felix Platter-Spital, Burgfelderstrasse 101, 4055 Basel, Switzerland

## Abstract

**Introduction:**

Optimal identification of subtle cognitive impairment in the primary care setting requires a very brief tool combining (a) patients’ subjective impairments, (b) cognitive testing, and (c) information from informants. The present study developed a new, very quick and easily administered case-finding tool combining these assessments (‘BrainCheck’) and tested the feasibility and validity of this instrument in two independent studies.

**Methods:**

We developed a case-finding tool comprised of patient-directed (a) questions about memory and depression and (b) clock drawing, and (c) the informant-directed 7-item version of the Informant Questionnaire on Cognitive Decline in the Elderly (IQCODE). Feasibility study: 52 general practitioners rated the feasibility and acceptance of the patient-directed tool. Validation study: An independent group of 288 Memory Clinic patients (mean ± SD age = 76.6 ± 7.9, education = 12.0 ± 2.6; 53.8% female) with diagnoses of mild cognitive impairment (n = 80), probable Alzheimer's disease (n = 185), or major depression (n = 23) and 126 demographically matched, cognitively healthy volunteer participants (age = 75.2 ± 8.8, education = 12.5 ± 2.7; 40% female) partook. All patient and healthy control participants were administered the patient-directed tool, and informants of 113 patient and 70 healthy control participants completed the very short IQCODE.

**Results:**

Feasibility study: General practitioners rated the patient-directed tool as highly feasible and acceptable. Validation study: A Classification and Regression Tree analysis generated an algorithm to categorize patient-directed data which resulted in a correct classification rate (CCR) of 81.2% (sensitivity = 83.0%, specificity = 79.4%). Critically, the CCR of the combined patient- and informant-directed instruments (BrainCheck) reached nearly 90% (that is 89.4%; sensitivity = 97.4%, specificity = 81.6%).

**Conclusion:**

A new and very brief instrument for general practitioners, ‘BrainCheck’, combined three sources of information deemed critical for effective case-finding (that is, patients’ subject impairments, cognitive testing, informant information) and resulted in a nearly 90% CCR. Thus, it provides a very efficient and valid tool to aid general practitioners in deciding whether patients with suspected cognitive impairments should be further evaluated or not (‘watchful waiting’).

## Introduction

Cognitive disorders are frequently underdiagnosed and, consequently, diseases such as dementia are undertreated [1]. The early identification of cognitive impairments is critical to initiate diagnostic procedures, since early diagnoses lead to optimal treatment and potentially improve prognoses and decrease morbidity. National task forces and academies have concluded that routine dementia screening – that is, population-based screening of individuals irrespective of the existence of cognitive complaints – cannot be recommended based on existing data, most notably screening tests’ mediocre diagnostic specificity [2-5]. Instead, speciality groups recommend a case-finding strategy whereby general practitioners (GPs) test patients with suspected or observable early signs or symptoms of cognitive impairment [6-10]. This strategy requires optimally sensitive and specific case-finding tools that are well suited to the primary care setting.

Many patient-directed cognitive or informant-based tools are available for case-finding in the primary care setting [4,6,11-16]. Lin and colleagues list 51 such tools in their comprehensive review [4]. Most were initially designed as screening tools, and thus the majority were evaluated in only one study relevant to the primary care setting (that is, 36/51 instruments; see ‘supplements’, ‘supplemental content’, ‘supplement. additional information on interventions’ in [4]). Of these tools, the best-studied *cognitive* (patient-directed) case-finding tool is the Mini-Mental Status Examination (MMSE) [17]. However, the MMSE suffers from low inter-rater reliability (IRR) and low sensitivity for mild impairment (for example [18]) and inappropriateness for primarily nonverbal forms of cognitive impairment (for example, mild cognitive impairment (MCI) and dementia in Parkinson disease [19]). The Montreal Cognitive Assessment is similar in design to the MMSE in that it assesses multiple cognitive domains and has a maximum of 30 points [20]. Compared with the MMSE, the Montreal Cognitive Assessment identified more MCI and Alzheimer’s dementia (AD) patients (sensitivity) but fewer healthy controls (specificity) [20]. Additional instruments include the Rowland Universal Dementia Assessment Scale [21] and the CANTABmobile (Cambridge Cognition, Cambridge, UK), a 10-minute to 15-minute screening instrument comprised of a visual paired associates learning test, the Geriatric Depression Scale, and assessment of activities of daily living. While these tools are valuable instruments to quickly assess global cognitive functioning, particularly in a memory clinic setting [22], their relatively long administration time and potential burden to the patient–GP relationship reduce their feasibility and acceptability in the primary care setting.

Shorter patient-directed cognitive tools that have likewise been well studied include the clock drawing test (CDT) [23], Mini-Cog [24], and the Memory Impairment Screen [6,11-16,25]. The CDT primarily assesses executive dysfunction and is less influenced by sociodemographic factors (for example, educational level, language) than other case-finding tools [14]. The CDT yields adequate sensitivity and specificity for dementia when administered on its own [14], which are improved when combined with other cognitive instruments that assess episodic memory functioning [26]. For example, the Mini-Cog combines the CDT with a three-item delayed word recall task [24]. Also the Mini-Cog can be administered very quickly (circa 3 minutes), has a high sensitivity and correct classification rate (CCR), and is less susceptible to low education and literacy than, for example, the MMSE [24,27]. The Memory Impairment Screen is a short cognitive case-finding tool that focuses purely on episodic memory functioning [25]. This four-item delayed and cued recall test can be quickly administered and performance is not significantly affected by demographic factors, but its sensitivity and specificity are inferior to, for example, Mini-Cog [13]. Thus, short instruments that combine executive and episodic memory assessments appear most promising for accurate case-finding in the GP setting.

The subjective impression of the patient’s informant (for example, family member) is a critical component of an accurate assessment of the patient’s cognitive and behavioral profiles [6,28,29], although this information may not always be available. Informants may notice changes in cognitive functioning not noticed by patients, especially when patients suffer from neurodegenerative syndromes associated with a lack of awareness [30]. One widely used informant-directed tool that is available in many different languages is the Informant Questionnaire on Cognitive Decline in the Elderly (IQCODE) [31,32]. This tool performs adequately in the primary care setting [4]. Moreover, a very short, seven-item version of the IQCODE is available, which distinguishes healthy older individuals from patients with AD and MCI with high correct classification rates (that is, 90.5% and 80.1%, respectively) [33]. The General Practitioner Assessment of Cognition (GPCOG) [28] was developed specifically as a GP case-finding tool and combines two critical sources of information: patient-directed cognitive testing (that is CDT, time orientation, a report of a recent event and a word recall test) and informant-directed questioning (that is, six informant questions asking whether the patient’s functioning has changed compared with ‘a few years ago’). Although the combination of two separate sources of information is assumed to bolster diagnostic accuracy compared with tests relying on one source of information, surprisingly the diagnostic accuracy of the GPCOG was comparable with that of the MMSE (that is, area under the receiver operating characteristic curve = 0.91 vs. 0.89, respectively) [28].

Cordell and colleagues recently suggested that case-finding tools can be further optimized by including questions on patient’s subjective functioning [6]. Indeed, questions on subjective cognitive impairments [34] and depression [35] are good predictors of the future development of, for example, MCI and dementia [36]. Moreover, these questions are easy to administer in the primary care setting as they typically correspond to the questions GPs pose during routine history-taking.

The emerging consensus regarding case-finding for patients with potential MCI and early dementia in the primary care setting is that it optimally requires the combination of patient-directed cognitive testing, informant information on patient functioning and patient information on subjective cognitive impairments [6]. However, to our knowledge, no case-finding tool combines these three components into a single tool that is feasible to administer in the primary care setting; that is, a tool which is very brief and nonthreatening. The first goal of this study was therefore to develop a very brief, user-friendly case-finding tool for primary care physicians that combines a patient-directed tool (that is, cognitive testing and subjective patient information) and an informant-directed tool (that is, subjective informant information). We then performed a study to obtain GPs’ judgments on the feasibility of the patient-directed instrument; that is, the instrument administered by GPs. Finally, we conducted a validation study to determine the optimal scoring criteria and corresponding CCR of the patient-directed instrument alone (applicable in situations in which no informant is available) and of the combined patient-directed and informant-directed tool; that is, BrainCheck. The informant-directed instrument (that is, very short IQCODE) was previously validated by Ehrensperger and colleagues [33].

## BrainCheck development

We describe the development of the BrainCheck instrument, followed by the feasibility and validation studies.

### Methods

BrainCheck is composed of patient-directed and informant-directed components.

#### Patient-directed instrument

A Swiss dementia task force, strengthened by an international advisory board (see Acknowledgements), was charged with developing a very brief (that is, applied in a few minutes) patient-directed instrument sensitive to subtle cognitive decline. The task force created the following yes/no questions about subjective memory performance (Q1 to Q3; cf. [37]) and depressive symptoms (Q4 and Q5 [38]):

1. Have you experienced a recent decline in your ability to memorize new things?
2. Have any of your friends or relatives made remarks about your worsened memory?
3. Do your memory or concentration problems affect your everyday life?
4. During the past month, have you often been bothered by feeling down, depressed or hopeless?
5. During the past month, have you often been bothered by little interest or pleasure in doing things?

The CDT [23] was included in the patient-directed instrument for the efficient assessment of cognitive, in particular executive, function. The CDT presents patients with a predrawn circle (10 cm diameter) and instructs them to ‘Please draw a clock with all the numbers and hands’ (no specific time or time limit specified). Once finished, patients are asked to ‘Write down in numbers the time shown on the clock you have just drawn, as it would appear on a timetable or TV guide.’ The following scoring criteria were used [26], each requiring a yes/no decision:

- CDT-1. Are exactly 12 numbers present?
- CDT-2. Is the number 12 correctly placed?
- CDT-3. Are there two distinguishable hands (length or thickness)?
- CDT-4. Does the time drawn correspond to the time written in numbers (±5 minutes)?

In addition, the following GP judgment of overall CDT accuracy was included:

- CDT-5. Was the clock, including ‘time in numbers’, perfect?

#### Informant-directed instrument

The very short and validated seven-item version of the IQCODE was selected as the informant-directed instrument [33]. Briefly, this tool requires informants to rate patients’ current cognitive abilities compared with 2 years earlier. Judgments are rated on a five-point scale from ‘much improved’ (1) to ‘much worse’ (5), with (3) representing ‘no change’. Informants are typically provided with the instructions and questions, and fill out the questionnaire on their own. The seven-item IQCODE includes the following items:

1. Remembering things about family and friends; for example, occupations, birthdays, addresses.
2. Remembering things that have happened recently.
3. Recalling conversations a few days later.
4. Remembering what day and month it is.
5. Remembering where to find things that have been put in a different place from usual.
6. Learning new things in general.
7. Handling financial matters; for example, pension, dealing with the bank.

The total score of the seven-item IQCODE is the mean of all items. A maximum of two missing answers was allowed, in which case the mean of the remaining items was used as the total score. This procedure was adopted to increase the generalizability of the present findings to the general practice setting, where informants do not always provide complete questionnaire data.

## Feasibility study

Following the construction of the patient-directed tool, a feasibility study was performed to acquire GPs' judgments on the feasibility and acceptability of the patient-directed tool; that is, the instrument which the GP administers. The patient-directed data collected here were not the focus of this feasibility study and were not analyzed to ascertain the validity of BrainCheck (see Validation study). However, inter-rater reliability of CDT scoring between GPs and expert memory clinic raters was calculated to determine the quality of the CDT scoring instructions.

The ethics committees at each recruiting site approved the study (Ethikkommission beider Basel; Comité d’Ethique Réhabilitation et Gériatrie – Psychiatrie, Hopitaux Universitaires de Genève; Kantonale Ethikkommission Bern; Commission d’Ethique de la Recherche Clinique, Lausanne; Ethikkommission des Kantons St. Gallen; Ethikkommission der beiden Stadtspitäler Triemli und Waid, Zurich), and all participants provided informed consent.

### Methods

#### Participants

Fifty-two GPs from the Basel and Lausanne regions of Switzerland agreed to assess between one and five consecutive patients fulfilling the following inclusion criteria: suspected cognitive problems (that is, reported by patient or informant or suspected by the GP), age ≥50 years, education ≥7 years, and fluent native speakers of German or French. Patients with severe auditory or visual impairments who could not complete the patient-directed instrument were excluded. In total, 184 patients were tested (60% women) with a mean age (± standard deviation) of 77.3 ± 9.1 years and a mean education of 11.5 ± 2.9 years.

#### Procedure

GPs administered the patient-directed tool according to a clinical record form. Following every administration of the patient-directed tool, GPs completed a questionnaire on the feasibility and acceptability of the patient-directed tool administration including judgments of wording and format, and patient's acceptance and understanding of the test. Questions were rated on a five-point scale: ‘not at all’ (1), ‘a little’ (2), ‘moderately’ (3), ‘quite a bit’ (4), ‘extremely’ (5). GPs were remunerated 50 CHF for each completed patient-directed tool questionnaire. Finally, GPs were invited to additionally provide their *global feedback* on the feasibility and acceptability of the patient-directed tool overall on a five-point scale: ‘strongly disagree’ (1), ‘disagree’ (2), ‘neither agree nor disagree’ (3), ‘agree’ (4), ‘strongly agree’ (5).

#### Statistical analyses

GPs’ judgments on the feasibility and acceptability of the patient-directed instrument were summarized descriptively. The IRRs of CDT scoring between the GPs and expert memory clinic raters were assessed in exploratory analyses.

### Results

Fifty-two GPs administered the patient-directed tool to 184 patients in response to memory complaints by the patient (65.8%) and/or by the informant (41.3%) and/or based on the GP’s suspicion of cognitive problems (42.9%). Each of these GPs rated the feasibility and acceptability of the instrument following every test administration.

These GPs judged the patient-directed questions to be well accepted (4.63 ± 0.73) (mean ± standard deviation based on the ratings ‘not at all’ (1), ‘a little’ (2), ‘moderately’ (3), ‘quite a bit’ (4), ‘extremely’ (5)) and understood (4.41 ± 0.83) by the patients, and the CDT to be acceptable to patients (4.57 ± 0.74).

Forty-nine of the 52 GPs additionally provided global feedback on the tool. They were satisfied with the patient-directed instrument (3.94 ± 0.83) (rated ‘strongly disagree’ (1), ‘disagree’ (2), ‘neither agree nor disagree’ (3), ‘agree’ (4), ‘strongly agree’ (5)). They considered the tool to be helpful (3.80 ± 0.91), with clearly worded questions (4.59 ± 0.67), in a format suited to the answers patients provide (4.25 ± 0.76) and economically suited to the healthcare system (4.41 ± 0.91). Of these physicians, 81.6% stated that they would use the tool if it was shown to be reliable and valid (4.02 ± 1.16).

The CDT scoring IRR between the GPs and expert raters in the feasibility study was κ = 0.50 to 0.69 for the five CDT criteria (all *P* >0.0001), indicating moderate agreement (CDT-1, κ **=** 0.69; CDT-2, κ = 0.50; CDT-3, κ = 0.59; CDT-4, κ = 0.53; CDT-5, κ = 0.50). Based on these results and verbal feedback from GPs in the feasibility study, refined CDT scoring instructions were created for the final version of BrainCheck. These revised CDT scoring instructions included a list of perfect clock criteria and additional guidance on test administration and scoring for GPs.

## Validation study

### Methods

The validation study aimed to determine the optimal score criteria for the patient-directed instrument alone (for patients with no informant) and the combined BrainCheck instrument, and their corresponding CCRs for cognitively healthy participants versus patients with cognitive symptoms. Ethics committees at all study sites (see Feasibility study) approved this study, and all participants provided informed consent.

#### Participants

All patients who participated in the validation study had been referred by their GPs to the memory clinics in Basel, Geneva, Berne, Lausanne, St. Gallen and Zurich. This patient sample was independent from patients who participated in the feasibility study. All patient-directed data for the validation study were then collected at the respective memory clinic. All patients received a comprehensive dementia work-up at the participating site that included comprehensive neuropsychological testing, an interview with the informant, an internal medical and neurological examination, psychopathological status and magnetic resonance brain imaging followed by an interdisciplinary diagnosis conference. All diagnosing clinicians were blind to patients’ BrainCheck results (the STARD initiative [39]). MCI was diagnosed according to Winblad and colleagues’ criteria [40]: deficits (score <–1.28 of demographically-adjusted *z* score) in one or more cognitive domain (that is, attention, executive functioning, memory, language, visuospatial functioning, praxia and gnosis) that represent a decline from an earlier level of cognitive functioning, but which were not severe enough to fulfill *Diagnostic and Statistical Manual of Mental Disorders*, 4th edition (DSM-IV) criteria for dementia (that is, intact activities of daily living as reported by next of kin). Dementia and major depression were diagnosed according to the DSM-IV criteria. The inclusion criteria for patients were: complete results from all aforementioned examinations; diagnoses of MCI [40], dementia (DSM-IV [41]) or major depression (DSM-IV [41]); age ≥50 years; MMSE [17] score ≥20/30; a minimum 7 years of education; and fluent German or French language proficiency. The exclusion criterion was the existence of sensory deficits that prohibited administration of BrainCheck.

Patient-directed data were gathered from 126 healthy older participants of an ongoing longitudinal study at the Memory Clinic in Basel; that is, the Basel Study on the Elderly [36,42]. These individuals had been examined with comprehensive neuropsychological testing and a thorough medical questionnaire. All individuals who fulfilled the following inclusion criteria were included in the present analyses: cognitively, neurologically and psychiatrically healthy; no high fever lasting longer than 1 week within the last week; no chronic pain; no full anesthesia within the last 3 months; fluent German language proficiency; and comparable age and education levels with the patient sample (see Table 1, entire sample).

**Table 1:** **Demographic characteristics of healthy individuals and patients in the validation study**

		**Cognitively impaired groups**			***P*** **value, NC vs. patients**
	**NC**	**Mild cognitive impairment**	**Dementia**	**Major depression**	
**Entire sample**					
*n*	126	80	185	23	
Female	50 (40)	44 (55)	99 (54)	12 (52)	0.008^a^
Age (years)	75.2 ± 8.8	74.9 ± 8.1	78.5 ± 6.5	67.4 ± 9.6	n.s.^b^
Education (years)	12.5 ± 2.7	12.2 ± 2.5	11.9 ± 2.7	12.4 ± 2.7	n.s.^b^
MMSE	28.9 ± 1.2	27.1 ± 2.2	24.2 ± 2.5	27.9 ± 1.8	0.001^b^
**BrainCheck subsample**					
*n*	70	21	86	6	
Female	20 (29)	12 (57)	44 (51)	4 (67)	0.001^a^
Age (years)	77.2 ± 8.9	75.3 ± 7.5	78.4 ± 6.3	68.2 ± 11.4	n.s.^b^
Education (years)	12.5 ± 2.9	11.6 ± 2.1	11.7 ± 2.6	11.2 ± 1.9	n.s.^b^
MMSE	28.6 ± 1.2	27.5 ± 1.8	24.4 ± 2.5	27.0 ± 1.9	0.001^b^

*Data presented as n (%) or mean ±* standard deviation. MMSE, Mini-Mental Status Examination; NC, healthy individuals. ^a^Chi-square test. ^b^ *t* test.

Seven-item IQCODE data were available from native German-speaking subgroups of 70 normal control participants and 115 patients. IQCODE data for two AD patients were excluded because of three missing items, resulting in 113 patients and 70 controls with comparable age and education levels (see Table 1, BrainCheck subsample).

#### Procedure

At each memory clinic, the patient-directed instrument was administered prior to the clinical neuropsychological battery by trained clinicians who neither conducted the clinical neuropsychological examination nor took part in any interdisciplinary diagnosis conference. Administration was standardized between memory clinics and conducted according to the Swiss consensus guidelines [43]. The 16-item German IQCODE was administered to informants in the BrainCheck subsample according to standardized procedures [44], and data from the seven-item form (that is, mean score [33]) were used in the present analyses.

#### Statistical analyses

The IRRs of CDT scoring between clinicians at the six memory clinics and expert raters at the Basel memory clinic were assessed in exploratory analyses.

For the following analyses, please note that the Memory Clinic diagnosis of healthy control or patient represented the criterion or gold standard against which the BrainCheck data were evaluated.

A classification and regression tree (CART) analysis was conducted to separate healthy controls from patients based on the patient-directed data. In its first step, CART identifies that variable which best discriminates between two groups, here between patients and control participants. If this variable is categorical, each value produces a branch of a tree. For each branch, the variable that best discriminates the subset of participants on this branch is determined, generating additional branches (for categorical variables), and so on. This process proceeds until improvements in fit fall below an *a priori* determined complexity parameter of 1%. The resulting decision rule proved to be stable in 10,000 bootstrap samples. Both the CART and bootstrapping analyses were performed in R software [45] using the R-package rpart. The resulting algorithm generates a case-finding result of either further evaluation (based on the probability that score X belongs to the present Memory Clinic patient distribution) or watchful waiting (based on the probability that score X belongs to the present healthy control distribution) based on the patient-directed instrument data alone.

We attempted to model the combined patient-directed and informant-directed data using two independent approaches: traditional logistic regression and the CART approach described above. Both approaches failed to adequately model the combined data, most probably because of higher-order interactions in the data associated with highly parameterized models. A decision algorithm was therefore developed by generating a contingency table of the patient-directed predictor variables identified by the CART analysis and the group variable (healthy participant vs. patient). Homogeneous subgroups consisting of either a high or a low proportion of patients were identified, corresponding to a preliminary classification. This preliminary classification was extended to include informant data from the seven-item IQCODE by determining separate optimal seven-item IQCODE mean cutoff scores for each homogeneous subgroup via receiver operating characteristic (curve) analyses modeling optimal CCRs. The ensuing decision rules were evaluated in 10,000 bootstrap replicates [46] to estimate the variability of their sensitivities and specificities.

### Results

CDT scores between memory clinic clinicians who regularly perform CDTs and the expert raters were comparable (IRR κ = 0.69 to 0.90; *P* >0.0001) (CDT-1, κ = 0.90; CDT-2, κ = 0.85; CDT-3, κ = 0.69; CDT-4, κ = 0.78; CDT-5, κ = 0.75).

A CART analysis was applied to the five questions (Q1 to Q5) and five CDT scoring criteria (CDT1 to CDT5) of the patient-directed tool to derive an algorithm that classified the maximum proportion of healthy participants into the watchful waiting group and the maximum proportion of patients into the further examination group. Individual item scores, as opposed to mean scores on Q1 to Q5 and CDT, were analyzed to determine which individual items contributed best to diagnostic discriminability. This procedure enabled us to drop inferior items, thereby maximizing BrainCheck’s efficiency and diagnostic accuracy.

**Figure 1:**
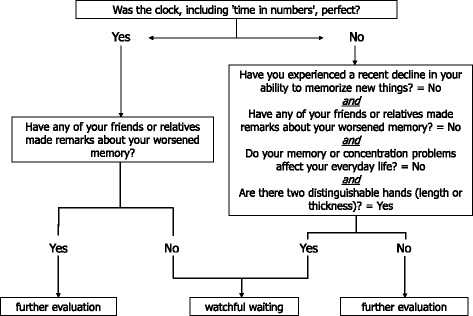
**Decision algorithm for the patient-directed tool.** Sensitivity = 83.0%, specificity = 79.4%, correct classification rate = 81.2%.

The CART selected four questions (Q1 to Q3, Q5) and two CDT scoring criteria (CDT-3, CDT-5) in the final model. The top of the classification tree is occupied by CDT-5 (perfect CDT), reflecting the largest weight in discriminating healthy individuals from patients. A middle level of the classification tree was occupied by the subjective questions on memory functioning (Q1 to Q3) and CDT-3 (‘two distinguishable clock hands?’). A final level of the tree was occupied by Q5 on depressed mood (‘little interest or pleasure in doing things?’). Since Q5 added an additional level of complexity to the classification algorithm, CCRs were calculated for classification algorithms with and without Q5 to determine whether classification complexity could be reduced without sacrificing CCR. These analyses revealed comparable CCRs with and without Q5 (difference: 1.3%); Q5 was therefore eliminated. The final algorithm included the three memory-related questions and two CDT items (see Figure 1). The application of this algorithm to the entire sample resulted in a high sensitivity (83.0%) and specificity (79.4%), with an overall CCR of 81.2%.

**Figure 2:**
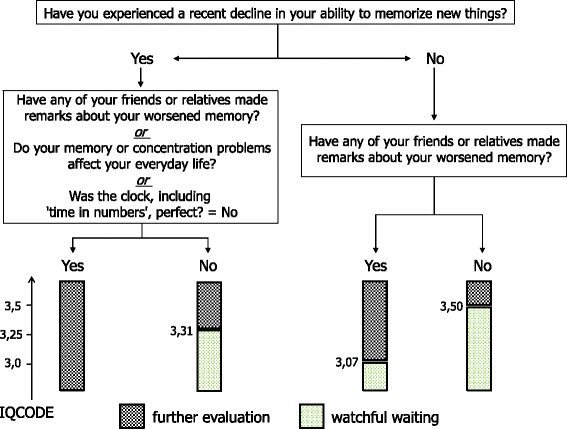
**Patient-directed and informant-directed case-finding (BrainCheck) decision algorithm.** Sensitivity = 97.4%, specificity =81.6%, correct classification rate = 89.4%. IQCODE, Informant Questionnaire on Cognitive Decline in the Elderly.

The combined patient-directed and informant-directed data were analyzed next (see Methods). The mean seven-item IQCODE score was used, whereby one missing item (17 patients, eight control participants) or maximally two missing items (three patients, zero control participants) were allowed (percentage of complete datasets: 83% for patients, 89% for control participants). A six-way contingency table with the five patient-directed variables identified by the CART analysis and the group variable (healthy participant vs. patient) demonstrated that the CDT-3 variable had no effect on the categorization of the BrainCheck sample into healthy individuals versus patients. The CDT-3 variable was therefore removed from the equation, and a second, five-way contingency table was generated with the four remaining patient-directed variables and the group variable. This contingency table revealed four homogeneous subgroups, corresponding to a preliminary classification. Receiver operating characteristic (curve) analyses for each of these homogeneous subgroups determined the IQCODE cutoff score (IQCODE_cut) that optimally categorized individuals. Thus, depending on the results of the patient-directed questions, different seven-item IQCODE cutoff scores were used to determine whether watchful waiting or further evaluation was indicated, where values exceeding the cutoff score indicate further evaluation (see Figure 2). Logically, further evaluation would be indicated when scores on both the patient-directed and informant-directed instruments would be deficient. However, this situation never occurred in the present sample. We therefore added this rule based on the aforementioned logic. The final BrainCheck decision rules are thus as follows:

1. If patient-directed instrument = not normal and IQCODE = not normal (that is, IQCODE mean ≥3.29), then BrainCheck = ‘further evaluation indicated’.
2. If Q1 = no & Q2 = yes, then use IQCODE_cut = 3.07.
3. If Q1 = no & Q2 = no, then use IQCODE_cut = 3.50.
4. If Q1 = yes & Q2 = no & Q3 = no & C5 = yes, then use IQCODE_cut = 3.31.
5. If Q1 = yes & not (Q2 = no & Q3 = no & C5 = yes), then ‘further evaluation indicated’.

The application of these rules to the BrainCheck sample resulted in a sensitivity of 97.3%, a specificity of 81.4%, and a CCR of 89.35%. The classification performance following 10,000 bootstrap replicates resulted in a mean sensitivity of 97.4% (quartile range: 96.4 to 98.3%), a mean specificity of 81.6% (quartile range: 78.4 to 84.6%) and a CCR of 89.4% (see Table 2).

**Table 2:** **Diagnostic discriminability of the individual and combined (BrainCheck) patient-directed and informant-directed screening instruments in the BrainCheck subsample**

	**Sensitivity (%)**	**Specificity (%)**	**CCR (%)**
Patient-directed instrument	85.8	74.3	79.9
Informant-directed instrument	81.4	75.7	78.6
BrainCheck	97.4	81.6	89.4

CCR, correct classification rate.

## Discussion

The present study developed and validated BrainCheck as a new, very short (that is about 3-minute) case-finding tool for cognitive disturbances combining direct cognitive testing, patients’ subjective impressions of their cognitive functioning and informant information. The feasibility study showed that GPs judged the administration of the patient-directed instrument to be feasible (that is, time efficient) and acceptable. The BrainCheck’s CCR reached nearly 90% in the validation study. The items with the greatest discriminatory power were questions on subjective (patient) and observed (informant) memory and executive functioning and the results of cognitive testing (that is, CDT). We therefore recommend the BrainCheck as a case-finding tool, because it meets the challenge of combining patient-directed cognitive testing, informant information on patient functioning and patient information on subjective cognitive impairments [6] and provides a high CCR. Because we realize that informants may not always be available to provide information on patients, we additionally determined the optimal scoring criteria and diagnostic accuracy of the patient-directed instrument alone. These analyses were performed on the largest dataset at our disposal in order to provide GPs with the most robust and reliable findings. These analyses revealed that the patient-directed instrument data correctly classified 81% of the BrainCheck validation sample, suggesting that it can be administered on its own when no informant is available. Critically, the patients in both validation samples were very mildly impaired (that is, MMSE = 25.3 ± 2.8, entire sample; MMSE = 25.1 ± 2.6, BrainCheck subsample), suggesting that this case-finding tool can detect subtle cognitive impairments in a primary care setting.

Cordell and colleagues recommended combining patient-directed and informant-directed case-finding questions at older individuals’ annual wellness visits when signs or symptoms of cognitive dysfunction are present [6]. Moreover, the new research diagnostic criteria for MCI recommend querying informants about patients’ cognitive functioning and the extent to which patients’ cognitive functioning has declined [47], and research criteria for probable AD recommend the acquisition of informant information on patients’ cognitive functioning [48]. Informant information is critical as family members may be more competent in judging changes in patients’ cognitive functioning in syndromes associated with an early lack of awareness; for example, AD [30]. Moreover, informant-based case-finding tools are obviously less confrontational for patients than direct cognitive testing.

The CDT was adopted because it is very brief and easy to administer and has acceptable sensitivity and specificity in differentiating healthy participants from those with cognitive impairments [14]. However, IRR analyses showed poor correspondence between the GPs and expert raters, in line with previous findings [49]. In response to this finding, we developed detailed scoring criteria for use in the GP setting. Two CDT variables – whether two distinguishable hands are present and whether the clock is perfect – were among the best discriminators between healthy participants and patients based on patient-directed data alone, whereas only the whether the clock is perfect variable survived in the BrainCheck algorithm. Similarly, Scanlan and colleagues recommended that untrained examiners use the rating normal CDT versus abnormal CDT for the classification of healthy versus demented individuals [50] (see also [13]). We note that 32% of healthy participants in the patient-directed sample drew imperfect clocks; it is therefore advisable to combine the CDT with other case-finding instruments for cognitive impairment.

Case-finding tools similar to the patient-directed and informant-directed components of BrainCheck exist (see Introduction and [4]), although to our knowledge no published tool combines the three sources of information deemed critical for optimal case-finding (that is, cognitive testing of patient, informant information, patients’ subjective impressions of their cognitive functioning) into a single, very short tool [6]. Perhaps the most similar tool in content and length is the GPCOG, which combines patient-directed cognitive testing and six informant questions [28]. In a validation study, Brodaty and colleagues studied 283 individuals from the community who complained of memory impairment [28]. The GPCOG was administered by local GPs, and the diagnostic gold standard was defined as memory clinic diagnoses. In this sample, the patient-directed GPCOG’s sensitivity and specificity were 82% and 70%, compared with 89% sensitivity and 66% specificity for the informant-directed GPCOG. The patterns of sensitivity and specificity in the GPCOG and BrainCheck patient-directed and informant-directed tools were thus similar, with greater sensitivity than specificity and overall comparable diagnostic accuracies across the two sources of data. The higher diagnostic accuracy for BrainCheck compared with GPCOG most probably originates from the nature of the present sample: memory clinic patients are more likely to have a positive diagnosis, and the healthy individuals participating in research studies are more likely to have a negative diagnosis compared with community-based residents. As the MMSE was used to select patients for the present validation study, a direct comparison of MMSE and BrainCheck sensitivity and specificity rates in the present sample is not warranted. Both the GPCOG and BrainCheck are thus brief, encompass multiple domains and demonstrate good diagnostic accuracy. BrainCheck’s, but not GPCOG’s, demonstrably superior diagnostic accuracy compared with the MMSE may be accounted for by the greater variability in MMSE administration methods across different GP practices in Brodaty and colleagues’ study [28] and BrainCheck’s inclusion of information on the patient’s subjective cognitive functioning.

There are three caveats to the present study. First, as noted above, the BrainCheck CCR is potentially artificially inflated by an inestimable magnitude since patients referred to a memory clinic more probably represent a subset of primary care patients with more cognitive impairment, and optimally healthy participants from the validation study are those that are less likely to be screened by GPs. Second, as with any validation study, we note that external validation, particularly in the GP setting, is required to confirm the performance levels of the patient-directed instrument alone and BrainCheck. We note that correct classification rates from studies in the GP setting are generally lower than those obtained from memory clinic samples. Finally, we note that our validation study sample included too few patients with depression to reliably test the validity of case-finding questions about this disorder.

## Conclusions

**Figure 3:**
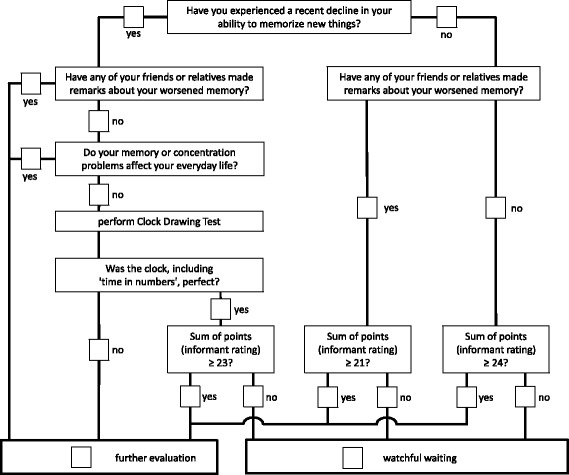
**Simplified, paper-and-pencil flowchart version of BrainCheck modified for optimally efficient administration.** In contrast to the decision algorithm (cf. Figures 1, 2), this simplified version requires the IQCODE sum. This version can therefore only be used when complete IQCODE data are available (that is, no missing data). IQCODE, Informant Questionnaire on Cognitive Decline in the Elderly.

There are several advantages to the new BrainCheck case-finding tool. First, there is no formal episodic memory task, which is time consuming and frequently frustrates patients and possibly GPs. Second, the patient-directed instrument can be administered in a very short time (that is, circa 3 minutes). Third, the seven-item IQCODE may be administered by a nurse, the physician's assistant or other trained professionals, saving the GP additional time. We provide decision algorithms that enable GPs to determine whether further evaluation or watchful waiting is indicated; that is, repeated screening in 6 to 12 months. When such a patient scores within the range of the present Memory Clinic patient sample, then the algorithm recommends further evaluation since an underlying syndrome is more probable. If the GP’s patient with suspected cognitive symptoms scores within the range of the present control sample, then the algorithm recommends watchful waiting; that is, the longitudinal observation of suspected cognitive symptoms. Lastly, while the BrainCheck decision algorithm is complex, we developed an easy-to-use App for the iPhone and iPad (Android app under development) for GPs and other healthcare service providers for a one-time nominal charge that automatically calculates the BrainCheck results. We also offer a paper-and-pencil version of the BrainCheck free of charge. The paper-and-pencil version has been modified to be optimally efficient, as it includes only those questions necessary to derive a decision (see Figure 3). In summary, BrainCheck meets criteria for an efficient and effective routine case-finding tool for primary care patients with suspected cognitive symptoms: it is brief, simple to use, sensitive and specific.

## Abbreviations

AD: Alzheimer’s dementia
CART: classification and regression tree
CCR: correct classification rate
CDT: clock drawing test
DSM-IV: *Diagnostic and Statistical Manual of Mental Disorders*, 4th edition
GP: general practitioner
GPCOG: General Practitioner Assessment of Cognition
IQCODE: Informant Questionnaire on Cognitive Decline in the Elderly
IQCODE_cut: cutoff score of the Informant Questionnaire on Cognitive Decline in the Elderly
IRR: inter-rater reliability
MCI: mild cognitive impairment
MMSE: Mini-Mental State Examination
